# Voice, Song and Speech

**Published:** 1885-07

**Authors:** 


VOICE, SONG AND SPEECH: A PRACTICAL GUIDE FOR SINGERS AND SPEAKERS; FROM THE COMBINED VIEW OF VOCAL SURGEON AND VOICE TRAINER. *By* LENNOX BROWNE, F. R. C. S., Ed., AND EMIL BEHNKE, *Teacher of Voice Production, etc. With numerous Illustrations. 8vo., cloth. pp. XV—322. New York:* G. P. PUTNAM'S SONS, *1884; Chicago:* JANSEN, MCCLURG & CO.

The value of this work is greater on account of the rarity of such treatises. Then it is indispensable to voice trainers and to physicians as well. Its numerous plates, including several photographs of the vocal cords, are well executed, and the whole volume is made up in the English high style with a quantity of blank space. As a book of reference it is a valuable addition to any library. And it will convey to any physician a good deal of information for which he may often vainly be called upon by the operatic class. The latter chapters treat of the Ailment of the Voice Uses, and of Defects of Speech—Stammering, etc. H. D. V.

